# Training Pre-Service Early Childhood Educators in Physical Activity (TEACH): Protocol for a Quasi-Experimental Study

**DOI:** 10.3390/ijerph19073890

**Published:** 2022-03-24

**Authors:** Patricia Tucker, Brianne A. Bruijns, Kristi B. Adamo, Shauna M. Burke, Valerie Carson, Rachel Heydon, Jennifer D. Irwin, Andrew M. Johnson, Patti-Jean Naylor, Brian W. Timmons, Leigh M. Vanderloo

**Affiliations:** 1 School of Occupational Therapy, Faculty of Health Sciences, Elborn College, Western University, London, ON N6G 1H1, Canada; 2 Health and Rehabilitation Sciences Program, Faculty of Health Sciences, Elborn College, Western University, London, ON N6G 1H1, Canada; bbruijns@uwo.ca; 3 School of Human Kinetics, Faculty of Health Sciences, University of Ottawa, Ottawa, ON K1N 6N5, Canada; kadamo@uottawa.ca; 4 School of Health Studies, Faculty of Health Sciences, Western University, London, ON N6A 3K7, Canada; sburke9@uwo.ca (S.M.B.); jenirwin@uwo.ca (J.D.I.); ajohnson@uwo.ca (A.M.J.); 5 Faculty of Kinesiology, Sport, and Recreation, University of Alberta, Edmonton, AB T6G 2H9, Canada; vlcarson@ualberta.ca; 6 Faculty of Education, Western University, London, ON N6G 1G7, Canada; rheydon@uwo.ca; 7 School of Exercise Science, Physical and Health Education, University of Victoria, Victoria, BC V8P 5C2, Canada; pjnaylor@uvic.ca; 8 Child Health and Exercise Medicine Program, McMaster University, Hamilton, ON L8S 4L8, Canada; timmonbw@mcmaster.ca; 9 Child Health and Evaluative Science, Hospital for Sick Children, 555 University Ave., Toronto, ON N5G 1X8, Canada; lvande32@uwo.ca

**Keywords:** physical activity, early childhood educator, sedentary behaviour, e-Learning

## Abstract

Background: Early childhood educators (ECEs) influence young children’s early uptake of positive health behaviours in childcare settings and serve as important daytime role models. As such, it is imperative that post-secondary early childhood education programs provide students with the foundational knowledge and professional training required to confidently facilitate quality active play opportunities for young children. The primary objective of the *Training pre-service EArly CHildhood educators in physical activity (TEACH) study* is to develop and implement an e-Learning course in physical activity and sedentary behaviour to facilitate improvements in: pre-service ECEs’ self-efficacy and knowledge to lead physical activity and outdoor play opportunities and minimize sedentary behaviours in childcare. This study will also explore pre-service ECEs’ behavioural intention and perceived control to promote physical activity and outdoor play, and minimize sedentary behaviour in childcare, and the implementation of the e-Learning course. Methods/Design: A mixed-methods quasi-experimental design with three data collection time points (baseline, post-course completion, 3-month follow-up) will be employed to test the e-Learning course in early childhood education programs (*n* = 18; 9 experimental, 9 comparison) across Canada. Pre-service ECEs enrolled in colleges/universities assigned to the experimental group will be required to complete a 4-module e-Learning course, while programs in the comparison group will maintain their typical curriculum. Pre-service ECEs’ self-efficacy, knowledge, as well as behavioural intention and perceived behavioural control will be assessed via online surveys and module completion rates will be documented using website metrics. Group differences across timepoints will be assessed using linear mixed effects modelling and common themes will be identified through thematic analysis. Discussion: The TEACH study represents a novel, evidence-informed approach to address the existing gap in physical activity and sedentary behaviour-related education in Canadian post-secondary early childhood education programs. Moreover, e-Learning platforms, can be employed as an innovative, standardized, and scalable way to provide ECEs with consistent training across jurisdictions.

## 1. Background

Physical activity is a critical behaviour which supports young children’s (<5 years) physical, psychological, and academic development [1], while also enriching their lives in the here and now [2]. Despite its recognized benefits, physical activity levels have been noted to be varied, yet often low, in childcare centres [3]. For example, in one Canadian study, young children were reported to engage in only 1.54 min/h of moderate-to-vigorous physical activity (MVPA) while attending childcare [4]. Equally concerning, young children in childcare participate in high rates (~41 min/h) of sedentary time [5], which is associated with gains in adiposity and delayed cognitive development [6]. These compositional behaviours are worrisome as physical activity levels decline as children age [7], and, because attendance in childcare centres in Canada is high [8]; therefore, establishing health behaviours early in life is critical. 

In Canada, two-thirds of young children attend childcare [8], equating to almost 720,000 childcare spaces in centre-based facilities across the country, and over 280,000 children are enrolled in regulated home-based facilities. Further, children are spending, on average, 28 h per week in these settings [9]. With evidence showing that the childcare setting influences children’s physical activity behaviours [4], and the high reach and daily contact time with young children, the childcare environment is a prime setting through which to target the early years population.

For young children attending childcare, early childhood educators (ECEs) serve as their primary daytime role models; they are responsible for scheduling physical activity within their daily programming, while also facilitating active play. Therefore, these individuals have the potential to influence young children’s developmental experiences and day-to-day well-being [10]. With respect to daily programming, ECEs have a responsibility to support and engage young children in meaningful learning opportunities [11], such as physical activity, which nurture children’s holistic development and well-being [1,12,13]. Notwithstanding the benefits of physical activity during early childhood [1], opportunities for physical activity in childcare remain infrequent and undervalued [4,14,15,16].

Of particular concern, ECEs have communicated that they lack the self-efficacy needed to confidently facilitate physical activity for young children in childcare [17,18] and have acknowledged that this is primarily due to their limited professional training in physical activity domains, both during post-secondary education [19,20] and on-the-job [21,22]. A recent investigation of the physical activity knowledge, training, and self-efficacy among pre-service ECEs (*n* = 1292) enrolled at 61 Canadian colleges/universities revealed that only ~30% reported receiving physical activity and screen-viewing-specific training in their program and a mere 15% were familiar with national movement guidelines for young children [19]. More positively, pre-service ECEs who reported undertaking physical activity education demonstrated greater self-efficacy to engage young children in appropriate physical activity opportunities [19]. According to Bandura’s Social Cognitive Theory [23], self-efficacy is one of the most important and robust determinants of behaviour. In support of this theory, a recent meta-analysis revealed that task self-efficacy was the strongest psychological predictor of teaching performance [24]. As such, to effectively prepare pre-service ECEs for their work post-graduation, it is critical that their pre-service education scaffolds their development of self-efficacy in relation to a wide range of teaching contexts, including facilitating physical activity and children’s development of physical literacy.

Required learning outcomes for early childhood education programs delineate that graduates must be able to “promote regular, healthy physical activity in all children” [25]; however, the reported gap in pre-service ECEs’ physical activity-related knowledge and education [19] makes it apparent that they may not be provided with adequate educational experiences to match these expectations. Further, sedentary behaviour-related education is largely overlooked in post-secondary early childhood education curricula [19]. Without proper education, it is unlikely that pre-service ECEs will include appropriate and engaging physical activity and motor skill experiences into their daily programming and schedules, and actively plan to minimize sedentary opportunities, once they enter into the childcare profession [10]. Intervention research within post-secondary early childhood education programs is needed to understand how introducing a tailored physical activity and sedentary behaviour curriculum for pre-service ECEs influences their physical activity and sedentary behaviour-related self-efficacy, knowledge, and intentions, and thus, better prepares them for supporting and facilitating more active behaviours among young children in childcare.

Physical activity interventions which include professional development/training show promise for supporting improved physical activity levels among young children. For example, Pate et al.’s intervention, which trained childcare teachers in structured and unstructured physical activity opportunities and integrating physical activity into lesson plans, resulted in improved physical activity levels among preschoolers [26]. A different study by Ward and colleagues found online training for ECEs to be effective at improving educators’ physical activity practices in childcare [27]. Moreover, these authors noted the ease with which online training could be integrated into ECEs’ training and the reach online training could have for this field. Finally, Bai and colleagues reported an increase in ECEs’ self-efficacy to engage children to be active in nature play following a professional development program [28]. Collectively, training for ECEs shows potential for improving physical activity opportunities in childcare settings. Despite this, few interventions have explored the impact of professional learning uniquely (it is frequently provided in combination with physical activity programming) and little is known about the impact of this training in pre-service programs (i.e., during ECEs’ post-secondary training).

### 1.1. Study Rationale

The need to support pre-service ECEs’ self-efficacy and knowledge related to promoting physically active behaviours among young children in childcare is clear. Supplementary professional learning opportunities related to children’s physical activity, movement skill development, and appropriate sedentary behaviours, is likely to increase their confidence and ability to facilitate supportive physical activity environments, policies, programming, and practices in the childcare setting [19]. The traditional in-person approach to professional learning for ECEs, while effective [29], is resource-intensive, and thus, lacks feasibility for large-scale implementation. Building on the success of past training for ECEs [29], this study will adopt an innovative approach by using an e-Learning platform to deliver tailored education to pre-service ECEs at the post-secondary level, addressing a critical gap in Canadian early childhood education curricula. The product will include an evidence-informed, easily accessed physical activity and sedentary behaviour course which can be integrated into early childhood education programs across Canada and adapted for use in global contexts.

### 1.2. Study Objective

The primary objectives of the Training pre-service EArly CHildhood educators in physical activity (TEACH) study are to examine if the e-Learning course increases pre-service ECEs’: 1. self-efficacy to engage children in physical activity and outdoor play, and minimize sedentary behaviour in childcare; and, 2. physical activity and sedentary behaviour-related knowledge. The secondary objectives of the study are to explore: 1. pre-service ECEs’ behavioural intention and perceived behavioural control to promote physical activity and outdoor play and minimize sedentary behaviour in childcare; and, 2. implementation (e.g., fidelity, feasibility, acceptability, pre-service ECEs’ and early childhood education program instructors’ experiences) and potential scalability (via the Consolidated Framework for Implementation Research (CFIR) checklist) of the e-Learning course. 

## 2. Methods

### 2.1. Study Design

A mixed-methods quasi-experiential design will be employed to test the effectiveness of the TEACH e-Learning course at improving pre-service ECEs’ physical activity and sedentary behaviour-related self-efficacy and knowledge. Purposefully selected English-speaking colleges/universities offering an early childhood education program in Canada (i.e., clusters) will be randomly assigned to the experimental (*n* = 9) or comparison (*n* = 9) condition. A 4-module e-Learning course (hosted on a secure learning management system platform) will be implemented by schools assigned to the experimental condition, while comparison schools will continue with their typical curriculum. Data will be collected at baseline, post-course completion, and 3-month follow-up. The study protocol and tools for the study have been approved by the Non-Medical Research Ethics Board (initial REB# 116816; January 2021) at Western University and ethics approval for the full-scale implementation will be sought prior to recruitment and data collection. Additional approval from participating college/university ethics boards will be completed, if requested

### 2.2. Theoretical Underpinning

In light of the importance of self-efficacy in influencing ECEs’ teaching practices [10,24], the proposed research will apply Bandura’s Social Cognitive Theory [23] to develop an e-Learning course that can facilitate improved physical activity and sedentary behaviour-related knowledge and self-efficacy among pre-service ECEs. Bandura highlights the importance of verbal persuasion and vicarious experiences to promote knowledge acquisition and self-efficacy development [23]; as such, the e-Learning course will incorporate a number of practical scenarios that will serve as observational models from which pre-service ECEs can learn, along with verbal cues to help important messages resonate with them. Scenario-based knowledge checks will also be included to test pre-service ECEs’ practical application of learned course content; according to Bandura, receiving positive reinforcement following correct responses (i.e., mastery experiences) will foster task self-efficacy development and reproduction of the behaviour [30].

In addition to the Social Cognitive Theory, we will also adopt Mayer’s Cognitive Theory of Multimedia Learning which denotes that a multimedia presentation of material through various information processing channels (e.g., auditory and visual) can combine to produce logical mental constructs that facilitate knowledge acquisition [31]. Each information receiving channel has a finite capacity to process (new) information and knowledge [32]; however, Mayer notes that when a message is delivered through multiple channels, this reinforcement of the message can enhance learning. As such, the e-Learning course will optimally challenge these information processing channels by utilizing text, voiceover, videos, and animations. 

### 2.3. Implementation Framework

The CFIR is commonly used to promote the translation of research to practice; as such, the TEACH study will follow constructs (*n* = 39) within this framework to ensure the research project is appropriately designed and scalable if proven effective. The CFIR comprises five domains: 1. intervention characteristics (i.e., how the intervention is designed to fit the target organization; e.g., completing a needs assessment and collaborating with those in the early childhood education field); 2. outer setting (i.e., the external political/social context within which an organization resides; e.g., the network of colleges/universities within each province/territory that follow the same accreditation standards); 3. inner setting (i.e., characteristics of the organization undergoing the intervention; e.g., early childhood education programs have communicated their wish to receive additional education in physical activity and sedentary behaviour domains); 4. individual characteristics (i.e., characteristics of individuals within the target organization; e.g., pre-service ECEs’ own interest in pursuing this type of education); and, 5. implementation process (i.e., how the intervention is implemented to promote fidelity and acceptance; e.g., partnering with early childhood education program staff and instructors to champion the intervention). Following these implementation constructs throughout the research project will act as a marker for intervention scalability.

### 2.4. e-Learning Course Development

The e-Learning course has been designed to be an engaging method of education, differing from the traditional approach of adding narration to a PowerPoint presentation. This will be achieved by enhancing learner engagement with the content (by utilizing within-module knowledge checks and interactive educational graphics) and provide acceleration of expertise through the use of video-based scenarios (i.e., increasing vicarious experiences, an important construct for building self-efficacy; as per the Social Cognitive Theory; [23,33]). Given the train-the-trainer approach (i.e., using experts to train staff) is widely problematized in professional learning [34], specifically due to the inability to address the diversity and individual needs of the trainee and push their knowledge and growth, the online, self-directed platform of the TEACH study e-Learning course will allow colleges/universities to incorporate the course when and how they see fit (while also allowing students dive deeper into specific content areas of interest to foster their professional development and learning goals). This approach also ensures consistency in training across the country and promotes practical future application in the childcare environment [35]. The course will be produced by a highly skilled e-Learning design team (including instructional and graphic designers), and we will follow Clark and Mayer’s Evidence-Based Guidelines for e-Learning Design to ensure the mode of delivery supports optimal learning among pre-service ECEs [33]. 

**Figure 1:**
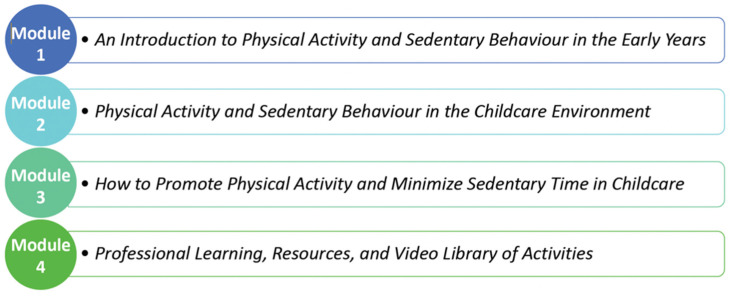
Breakdown of the TEACH study e-Learning course, by module.

Our team has developed specific e-Learning course content by way of a Delphi study, wherein international physical activity and sedentary behaviour experts (*n* = 26) proposed their top content areas to include in the course [36]. These content areas were pooled, and redistributed to the same experts, as well as 35 Canadian early childhood education experts, to be rated for their importance. From this generated content, the research team created associated learning objectives and module assessments (i.e., an online test to ensure the information was acquired) to complement accreditation standards for the early childhood education profession. The CFIR stresses the importance of tailoring interventions to the target population [37]; as such, we will solicit knowledge user input (from program instructors and pre-services ECEs) on module content and functionality throughout the e-Learning design phase. These collaborations will ensure the course content is contextually appropriate and the mode of delivery optimizes student learning. The e-Learning course will be hosted on a secure learning management system platform (Talent LMS), with a unique portal for each college/university, so pre-service ECEs can easily access it for completion in class or at home. To access the course, participants will simply create an account and login. Figure 1 outlines the proposed content of the 4-module e-Learning course.

### 2.5. Recruitment and Allocation

#### 2.5.1. Universities and Colleges with ECE Programs

To help ameliorate potential recruitment challenges and secure a diverse sample with representation from a number of Canadian provinces, we will draw on our established rapport with early childhood education post-secondary programs and will purposefully select colleges/universities (see sample size explanation below) and recruit their ECE programs to participate by emailing ECE program coordinators. Once 16 colleges agree to participate, allocation to the experimental and comparison conditions will occur. To safeguard the internal validity of the intervention, it is important to avoid randomly allocating individuals within each college to receive the training (or not), given the e-Learning course may be completed during pre-service ECEs’ class time and contamination may occur. As such, participating colleges/universities will act as the unit of randomization (i.e., clusters). Once consent has been obtained from the college/university representative (e.g., program coordinator/chair), the clusters will be stratified by province, college/university size, and college/university ECE program delivery mode (i.e., in-person, online, or blended learning). Blocked randomization will be performed to allocate clusters (1:1) using a computer-generated randomization tool (www.randomizer.org (accessed on 12 March 2022); the software will generate a number of 1 or 0) to either receive the training (experimental; “1”) or not (comparison; “0”).

#### 2.5.2. Pre-Service ECEs

The program coordinators and early childhood education instructors at participating colleges/universities will be given recruitment materials to distribute to their pre-service ECEs (including the link to the baseline survey). Prior to completing the baseline survey, pre-service ECEs must read the letter of information and consent. Voluntarily beginning the baseline survey will signify their consent to participate in the study. Programs and instructors will be invited to integrate the e-Learning course itself into their course (i.e., by providing class time to complete the course, if appropriate and available). Pre-service ECEs’ participation in the research study itself (i.e., surveys and interviews) will be voluntary (i.e., if pre-service ECEs opt not to participate, they can still access the e-Learning course if their instructor integrates this into class time). 

#### 2.5.3. ECE Instructors

Instructors at colleges/universities will be emailed a recruitment letter to ask if they would like to review the e-Learning course and provide their feedback about its content, functionality, and feasibility for the post-secondary early childhood education setting via a process evaluation survey and optional interview.

### 2.6. Inclusion and Exclusion Criteria

#### 2.6.1. Universities and Colleges with Early Childhood Education Programs

Canadian universities and colleges that offer an early childhood education program, where the pre-service ECEs are English-speaking, the program coordinator/chair agrees to the participation of their institution, and instructors are willing to participate, will be eligible to participate in this study.

#### 2.6.2. Pre-Service ECEs

English-speaking individuals who are enrolled in any early childhood education program (regardless of year of study or program type) within a participating Canadian university or college will be eligible to participate.

#### 2.6.3. ECE Instructors

English-speaking instructors who are employed within an early childhood education program allocated to the experimental group will be eligible to participate.

### 2.7. Sample Size

We used the ‘pwr’ package in R [38,39] to estimate the sample size for the analyses (Primary Objectives #1 and #2). These calculations suggest that we need 224 pre-service ECEs (112 per group) to detect a small-to-medium effect size (f^2^ = 0.053 or R^2^ = 0.05), with 2 groups (intervention and control) and 3 time-points (baseline, post-intervention, follow-up), 80% of the time, with an alpha of 0.025. As we will target college/university programs as units (clusters), the sample size will be adjusted to account for the clustering effect, where: D = design effect; *k* = anticipated cluster size (class size in this case); and *ρ* = the intra-cluster correlation coefficient, a measure of the degree of homogeneity among cluster subjects for a particular outcome investigated.

D = 1 + (*k* − 1) *ρ* = 1 + (164 − 1) (0.05) = 9.15

We estimate an average class size of 164 pre-service ECEs (based on our nationwide study of pre-service ECEs from 61 colleges; [19]). Based on a preliminary scan of early childhood education programs in Canada, programs across the country have comparatively similar curriculum; therefore, the intra-cluster correlation coefficient will be lower. For the purpose of estimating this sample size, we assumed a value of 0.05. Thus, the design effect is 9.15, and the sample size needs to be inflated. Finally, the sample will be further adjusted to account for loss to follow-up at 3 months (~30% attrition rate); therefore, the final targeted sample size will be 112 × 9.15/0.70 = 1464 ECE students per group.

### 2.8. Intervention Conditions

#### 2.8.1. Experimental Condition

At colleges/universities assigned to the experimental condition, participating pre-service ECEs will be required to complete the 4-module e-Learning course (in-class or at home, up to the instructor’s discretion) within a 4-week timeframe.

#### 2.8.2. Comparison Condition

Colleges/universities assigned to the comparison group will continue their typical curriculum for the duration of the study. Upon completion of the study, students in the comparison group may opt to offer the e-Learning course to their pre-service ECEs. Pre-service ECEs in the comparison group will complete the same study instruments as those in the experimental group (with the exception of the process evaluation survey and interviews).

### 2.9. Primary Outcome Measures

Various tools will be used to assess the impact of the TEACH study on pre-service ECEs’ physical-activity and sedentary behaviour-related self-efficacy and knowledge. See Table 1 for a description of tools and administration.

#### 2.9.1. Pre-Service ECEs’ Physical Activity and Sedentary Behaviour-Related Self-Efficacy

Our research team systematically reviewed physical activity and sedentary behaviour-related self-efficacy tools for ECEs; based on the findings from this review, no such tool emerged from the literature to meet the specific needs of this project [40]. Consequently, we created a 31-item questionnaire to assess ECEs’ Confidence in Outdoor Movement, Physical Activity, and Sedentary and Screen behaviours (*ECE-COMPASS*) [40]. This questionnaire, created by our team using Bandura’s Guide for Constructing Self-Efficacy Scales [41], assesses task (21 items) and barrier (10 items) self-efficacy and showed high internal consistency (Cronbach’s alpha > 0.90 across task and barrier subscales) and modest temporal stability (test-retest statistics > 0.60) [40]. This survey will be administered online at baseline, post-intervention, and 3-month follow-up, and composite scores for task and barrier self-efficacy will be calculated.

#### 2.9.2. Pre-Service ECEs’ Physical Activity and Sedentary Behaviour-Related Knowledge

As no validated tool exists to examine pre-service ECEs’ physical activity or sedentary behaviour-specific knowledge, our team developed a tool to assess this outcome in our study participants by creating questions based upon e-Learning course content (Appendix A). This 22-item online survey will be administered at baseline, post-intervention, and 3-month follow-up, and a composite score will be generated. Psychometric properties will be analysed and reported.

### 2.10. Secondary Outcome Measures

#### 2.10.1. Demographic Characteristics

Participant demographics, including age, gender, ethnicity, college/university, year of study, and program type and delivery method, will be captured via an online survey at baseline. Participants’ self-reported physical activity and recreational screen-viewing levels, previous professional learning courses/webinars in physical activity or sedentary behaviour (i.e., number taken), interest in physical activity and sedentary behaviour education (5-point scale; 0 = not interested at all to 4 = very interested), and previous experience with e-Learning (yes or no) will also be captured at both baseline and post-intervention.

#### 2.10.2. Learning Management System Metrics

E-Learning course completion rates (percent), usage data (completion time per module), and pre-service ECE attrition will be explored within the secure module platform to examine program fidelity. Student answers for module knowledge assessments (i.e., the 12-question assessments completed before proceeding to the subsequent module) will also be examined to measure short-term retention of material. Colleges/universities will be asked to share whether they required students to complete the modules as a course deliverable (produce a certificate of completion). 

#### 2.10.3. Behavioural Intention and Perceived Behavioural Control Survey

The ECEs’ Movement Behavioural Intention and Perceived Control (ECE-MBIPC) questionnaire will be completed by pre-service ECEs at baseline, post-intervention, and 3-month follow-up to gather their perspectives regarding their attitudes toward promoting physical activity and minimizing sedentary behaviour in childcare. This 56-item tool, adapted from Gagné and Harnois [42], is based on the Theory of Planned Behaviour [43] and measures psychosocial variables, including behavioural intention (28 items) and perceived behavioural control (28 items) to promote physical activity and outdoor/risky play, minimize prolonged sedentary time, and avoid screen time in childcare. Seven behaviours are measured for each psychosocial variable, and each behaviour is measured using four items; composite scores will be calculated for each of the seven behaviours for both behavioural intention and perceived behavioural control. Reliability analyses for the ECE-MBIPC tool have demonstrated high internal consistency (Cronbach’s alpha > 0.85) and acceptable temporal stability (>0.70) [44].

#### 2.10.4. Process Evaluation Survey—Pre-Service ECEs

An online process evaluation survey (38 items) will be completed by participants in the experimental group to gather their perspectives on the acceptability (i.e., satisfaction with the course), compatibility (i.e., appropriateness of the course for integration in ECE curricula), usability of the e-Learning platform, complexity of the content, and perspectives regarding suggestions for improvement. The survey will be informed by the Evaluating e-Learning System Success (EESS) model [45] to capture e-Learning-specific perspectives, and additional questions will capture their perspectives on course content.

#### 2.10.5. Process Evaluation Survey—ECE Instructors

An online survey (23 items) will be completed by ECE instructors at each institution in the experimental group to gather their current teaching of physical activity and sedentary behaviour content (including which specific concepts are covered), their perspectives on the course design, content, and implementation, and the feasibility of integrating the e-Learning course into their curricula.

#### 2.10.6. Interviews

A sample of pre-service ECEs (*n* = ~24) and instructors (*n* = ~12) will be interviewed to gain in-depth feedback on the e-Learning course’s implementation, content, and delivery. All interviews will last 30–45 min and will be digitally recorded and transcribed verbatim. A semi-structured interview guide, with probes, will be used to direct the conversation (Appendix B). Interviews will gather pre- and in-service ECEs’ experiences with the e-Learning course (e.g., its functionality, challenges experienced completing the course, and characteristics of the course that best facilitated learning), as well as their perspectives about learning physical activity and sedentary behaviour-related content. Interviews with instructors will gather their perspectives on the e-Learning course content, its alignment with their current early childhood education curriculum, student engagement with the course, and any alterations that would improve the course.

#### 2.10.7. CFIR Checklist

To ensure our intervention is designed and implemented with specific consideration to each of the five domains within the CFIR (e.g., it is evidence-based, stakeholder-supported, and trialled prior to widespread implementation), a checklist, created from the 39 constructs of the CFIR, will be completed by research staff (Appendix C). The research team will document how each construct of the CFIR (e.g., intervention complexity and adaptability, implementation climate, fidelity) is satisfied throughout the intervention (i.e., using data from the needs assessment, the Delphi content development study, website metrics, process evaluation survey, and interviews). Completion of this checklist will be indicative of the scalability of the TEACH study to a wider population.

### 2.11. Pilot Testing

To ensure the e-Learning course is functional, easy to use, and has a reasonable time commitment, we will pilot test the course, first, with ~150 in-service ECEs (from across the country, recruited via social media), and second, with pre-service ECEs attending college/university in two provinces and one territory (~50 students). While the course is designed for the pre-service ECE population, pilot testing in a sample of in-service ECEs will be undertaken to ensure the course provides them with relevant and useful education to support ECEs’ programming of active opportunities in a variety of childcare settings (and if effective, to support future implementation with this population). Participants will complete the e-Learning course and will be asked to complete an online survey post-intervention to gather specific feedback on the course’s functionality and pre- and in-service ECEs’ satisfaction with the course content and design. Semi-structured interviews with pre-service (~8) and in-service ECEs (~8) will be conducted post-intervention to gather more in-depth information on their experiences with the course. Additionally, to explore preliminary efficacy of the intervention, a secondary objective of the pilot study will be to measure changes in participants’ physical activity and sedentary behaviour-related: 1. self-efficacy; 2. knowledge; and 3. behavioural intention and perceived behavioural control (via online survey at pre- and post-intervention). After identifying strengths and weaknesses of the e-Learning course, appropriate changes will be made prior to full-scale implementation. Pilot testing is currently underway, with full scale implementation targeted to begin in September 2022.

### 2.12. Data Analyses

Baseline characteristics of the pre-service ECEs and their physical activity programming offerings (based on the curriculum review) will be summarized descriptively. Group differences at baseline will be assessed by independent sample *t*-tests or chi-square tests, as appropriate. If applicable, group differences on baseline measures will be statistically controlled in subsequent analyses (e.g., gender, age, and amount (number of course hours and content areas covered) of physical activity and screen-viewing training provided by the college/university). 

#### 2.12.1. Primary Outcomes

Pre-service ECEs’ physical activity and sedentary behaviour-related self-efficacy and knowledge will be evaluated using two (self-efficacy, knowledge) linear mixed effects models wherein group and time are our primary fixed effects. Demographic variables will be used as predictors of pre-service ECEs’ self-efficacy, knowledge, and behavioural intention and perceived behavioural control in our analyses. Specifically, we will explore the extent to which province/territory predicts the dependent variables, and whether there is a significant effect of previous training, early childhood education program type, and mode of delivery. Based on previous research, the pre-service ECE population is largely female [19], so gender-based differences will not be explored. Finally, if baseline differences are observed, we will include student age, physical activity, and sedentary time as covariates in all models. 

#### 2.12.2. Secondary Outcomes

Behavioural intention and perceived behavioural control will be evaluated using a linear mixed effects model, with group and time as fixed effects. As was the case in our primary outcome, we will evaluate the extent to which early childhood education program type, and the province or territory in which the program is situated, impact on our prediction models—and will also include student age, physical activity, and sedentary time as model covariates, if appliable. For the process evaluation, website metrics, and CFIR checklist, data will be explored using descriptive statistics. Thematic analysis [46] will be used to analyse open-ended questions from the process evaluation surveys and the interview transcripts. In line with Guba and Lincoln’s recommendations, steps will be taken to ensure data trustworthiness (e.g., credibility, transferability, dependability, and confirmability; [47]).

## 3. Discussion

A large body of research supports that the childcare environment, inclusive of ECEs’ programming, practices, and facilitation behaviours, can substantially influence children’s activity levels in this setting [4,48,49,50]. However, a gap in physical activity and sedentary behaviour-specific education has been noted to limit pre- and in-service ECEs’ confidence and ability to lead physical activity opportunities in childcare [10,19]. Providing pre-service ECEs with physical activity and sedentary behaviour-related training using an e-Learning platform is an innovative way to reach a large number of pre-service ECEs, while also making it easier to integrate into pre-existing early childhood education curricula. As such, the TEACH study represents a novel approach to population health that has the potential to advance pre-service training for ECEs across Canada.

In the interest of creating an evidence-informed physical activity intervention, the TEACH study was developed on the basis of Bandura’s Social Cognitive Theory [23] and Mayer’s Cognitive Theory of Multimedia Learning [31] for students with a variety of different learning styles. In addition, the e-Learning course content areas were derived following a rigorous Delphi method [51] with input from both physical activity and early childhood education experts [36]. These approaches not only ensure that the e-Learning course covers theoretically driven, relevant, and up-to-date content for promoting healthy activity behaviours in childcare, but they also verify that the course content is presented in line with pedagogy in the early years. Considering the involvement of stakeholders throughout the intervention process is linked to more effective implementation and higher adoption of behaviour change interventions [52], the participation of early childhood education stakeholders from the beginning of the TEACH study sets the intervention up for greater success. Further, piloting the e-Learning course with both pre- and in-service ECEs, and gathering input on its functionality through the application of the EESS model, will ensure a high-quality e-Learning experience is provided to students. With more post-secondary institutions shifting toward online class offerings [53], the TEACH study will deliver a timely and fitting solution to address the curriculum gap in physical activity and sedentary behaviour within early childhood education programs.

Other interventions have been developed to better support ECEs in promoting active behaviours among young children [26,29]; however, this is the first study to target pre-service training via an online platform. Pate et al. [26] recently incorporated physical activity training for educators into a childcare intervention, and preschoolers increased their energetic physical activity by 4 min/day. Even more promising, a feasibility study with the educators involved in Pate and colleagues’ project (*n* = 17) showed that they reported higher knowledge and self-efficacy to lead physical activity opportunities following the intervention, while also communicating that they would use the knowledge they gained in future programming [54]. As such, there is great potential for training interventions to make a difference in the physical activity experiences offered in childcare settings. However, the effectiveness of such training at the pre-service level needs to be explored, as this is the best platform to target all future ECEs. 

## 4. Conclusions

Utilizing e-Learning platforms to complement pre-service ECEs’ post-secondary education is a forward-thinking approach to ensure future ECEs receive necessary health promotion education. If successful, ECE graduates will enter their profession with greater self-efficacy to engage young children in physical activity and reduce sedentary behaviours during childcare hours, which is likely to increase the quantity and quality of programming offered to children in their care. Moreover, offering pre-service ECEs this training online improves reach. If effective, we will translate the e-Learning course into French to easily be employed by colleges/universities across Canada, and will support its adoption in countries where movement guidelines for the early years are similar to Canada’s (e.g., Australia, New Zealand; [55,56]). This protocol paper offers a detailed account of the TEACH study and tools for future investigations which aim to increase ECEs’ physical activity-related knowledge and self-efficacy via e-Learning. The results of the TEACH study will be shared with early years physical activity researchers globally and disseminated to Canadian colleges/universities with early childhood education programs, early years policymakers, childcare staff and directors, as well as other key stakeholders to ensure those responsible for the programming and practices within the childcare environment have the knowledge necessary to make decisions in support of healthy active behaviours among young children.

## Figures and Tables

**Table 1:** Tools Used in the TEACH Study.

		Experimental Group			Comparison Group		
		Baseline	Post	3-Month Follow-Up	Baseline	Post	3-Month Follow-Up
Pilot Study							
Pilot Test Participants	Consent	X					
	Demographic Survey	X					
	Self-Efficacy Questionnaire	X	X				
	Knowledge Questionnaire	X	X				
	Behavioural Intention and Control Survey	X	X				
	Process Evaluation Survey		X				
	Interview		X				
Quasi-Experimental Study							
University/College	Consent	X			X		
	Curriculum Review	X			X		
Instructors	Consent	X					
	Process Evaluation Survey		X				
	Interview		X				
Pre-Service ECEs	Consent	X			X		
	Demographic Survey	X	X		X	X	
	Self-Efficacy Questionnaire	X	X	X	X	X	X
	Knowledge Questionnaire	X	X	X	X	X	X
	Behavioural Intention and Perceived Behavioural Control Survey	X	X	X	X	X	X
	Process Evaluation Survey		X				
	Interview		X				
Research Team	Website Metrics	X	X				
	CFIR Checklist	X	X	X	X	X	X

TEACH = Training pre-service EArly CHildhood educators in physical activity; ECE = early childhood educator; CFIR = Consolidated Framework for Implementation Research.

## Data Availability

Not applicable.

## Abbreviations

MVPA	Moderate-to vigorous-intensity physical activity
ECE	Early Childhood Education
EESS	Evaluating e-Learning System Success
TEACH	Training pre-service EArly CHildhood educators in physical activity
CFIR	Consolidated Framework for Implementation Research

## Appendix A

**Pre-Service ECEs’ Physical Activity and Sedentary Behaviour-Related** **Knowledge Survey—Sample**
The following questions will test your knowledge of the Canadian 24-h Movement Guidelines for the Early Years (0–4 years):
1. How many minutes of tummy time are infants (<1 year) recommended to engage in each day? ⚪ 10 min (1) ⚪ 20 min (2) ⚪ 30 min (3) ⚪ 40 min (4)
2. How many minutes of total physical activity (i.e., any intensity physical activity) are toddlers (1–2 years) and preschoolers (3–4 years) recommended to engage in each day? ⚪ 60 min (1) ⚪ 90 min (2) ⚪ 120 min (3) ⚪ 180 min (4)
3. How many minutes of moderate-to-vigorous physical activity (i.e., higher intensity physical activity) are preschoolers (3–4 years) recommended to engage in each day? ⚪ 30 min (1) ⚪ 60 min (2) ⚪ 90 min (3) ⚪ 120 min (4)
4. How many minutes of screen time should a 3-year-old be limited to each day? ⚪ 30 min (1) ⚪ 60 min (2) ⚪ 90 min (3) ⚪ 120 min (4)
5. How much good-quality sleep, including naps, should infants (4–11 months) get each day? ⚪ 10–13 h (1) ⚪ 11–14 h (2) ⚪ 12–16 h (3) ⚪ 14–17 h (4)
6. How much good-quality sleep, including naps, should toddlers (1–2 years) get each day? ⚪ 10–13 h (1) ⚪ 11–14 h (2) ⚪ 12–16 h (3) ⚪ 14–17 h (4)
The following questions will test your knowledge of research-based recommendations for physical activity and screen-viewing at childcare:
7. For full-day programs (8 h), what is the recommendation for preschoolers’ (3–4 years) physical activity while in care? ⚪ 60 min/day of total physical activity, 20 min of which is at a moderate-to-vigorous intensity (1) ⚪ 90 min/day of total physical activity, 30 min of which is at a moderate-to-vigorous intensity (2) ⚪ 120 min/day of total physical activity, 40 min of which is at a moderate-to-vigorous intensity (3) ⚪ 180 min/day of total physical activity, 60 min of which is at a moderate-to-vigorous intensity (4)
8. What is the recommendation for screen-viewing in childcare? ⚪ There are no screen-viewing limits (1) ⚪ Limit screen-viewing to 30 min/day (2) ⚪ Screen-viewing is only recommended for educational purposes (3) ⚪ Screen-viewing is not recommended (4)

**End of Block: Knowledge—Guidelines**

**Start of Block: Knowledge—Definitions**

The following questions will assess your knowledge of common terms related to physical activity and sedentary behaviour among young children.
9. Galloping, hopping, and jumping are examples of what type of fundamental movement skill? ⚪ Stability (1) ⚪ Locomotor (2) ⚪ Manipulative (3) ⚪ Isometric (4)
10. What is an example of a muscle and bone-strengthening activity? ⚪ Playing catch (1) ⚪ Kicking a ball (2) ⚪ Balancing on a bench (3) ⚪ Jumping rope (4)
11. The “motivation, confidence, physical competence, knowledge, and understanding to value and take responsibility for engagement in physical activities for life” is the definition of what? ⚪ Structured physical activity (1) ⚪ Active lifestyle (2) ⚪ Physical literacy (3) ⚪ Active play (4)
12. What type of play is “a form of gross motor or total body movement in which young children use energy in a fun and freely chosen manner”? ⚪ Outdoor play (1) ⚪ Loose parts play (2) ⚪ Risky play (3) ⚪ Active play (4)
13. What type of play invites curiosity by allowing children to play with everyday items (such as kitchen utensils and cardboard boxes) or natural elements (such as tree stumps or pebbles)? ⚪ Outdoor play (1) ⚪ Loose parts play (2) ⚪ Risky play (3) ⚪ Active play (4)
14. What practice can be used to limit sedentary time while waiting for the next activity or travelling to a different part of the classroom? ⚪ Active breaks (1) ⚪ Active learning (2) ⚪ Active transitions (3) ⚪ Active play (4)
15. What is not considered a category of risky play? ⚪ Play at heights (1) ⚪ Play with tools (2) ⚪ Play near elements (e.g., water, fire) (3) ⚪ Play with loose parts (4)

**End of Block: Knowledge—Definitions**

**Start of Block: Knowledge—Educator Behaviours**

The following questions will assess your knowledge of appropriate behaviours of early childhood educators regarding activity promotion in childcare.
16. Which of the following behaviours of early childhood educators does not promote physical activity? ⚪ Co-participating in activities (1) ⚪ Engaging in passive supervision during outdoor play (2) ⚪ Providing verbal prompts (3) ⚪ Role modelling active behaviours (4)
17. Which strategy does not encourage risky play? ⚪ Always helping (1) ⚪ Trusting the children (2) ⚪ Asking the right questions (3) ⚪ Making time for it (4)
18. When it comes to outdoor play, it is okay to move activities indoors if: ⚪ It is lightly raining or snowing (1) ⚪ I don’t feel like going outside (2) ⚪ The playground is wet (3) ⚪ There is severe weather (4)
19. When is it not appropriate to lead structured physical activities during outdoor play? ⚪ When showing children how to use equipment (1) ⚪ When children consistently settle into sedentary play (2) ⚪ When children are engaging in rough and tumble play (3) ⚪ When children say they are bored (4)
20. To make a throwing activity more challenging, you can: ⚪ Move the target closer (1) ⚪ Use a bigger ball (2) ⚪ Use a smaller target (3) ⚪ Use two hands (4)
21. According to the Active Play and Physical Literacy Everyday (APPLE) Model, what four elements can educators utilize to encourage their children’s development of physical literacy? ⚪ Play, Relationships, Environment, Engagement (1) ⚪ Play, Leadership, Environment, Skills Development (2) ⚪ Leadership, Relationships, Engagement, Supervision (3) ⚪ Environment, Relationships, Supervision, Skills Development (4)
22. Why is communicating with families about movement behaviours at childcare important? ⚪ To encourage them to buy active and outdoor clothing for your class (1) ⚪ So you can tell them what they’re doing wrong at home (2) ⚪ It can help them understand why movement is part of your programming and how they can support progress at home (3) ⚪ To justify your programming decisions and show them how much you know (4)

## Appendix B

**Training Pre-Service EArly CHildhood Educators in Physical Activity:** **The TEACH Pilot Study** **Sample Interview Guide for ECE Students**

Thank you for volunteering to participate in this interview. Today we will discuss your thoughts and experiences of the recently implemented TEACH intervention; an e-Learning physical activity training program for [Early Childhood Education (ECE) students in Canadian colleges/universities]. Specifically, we are looking to gather your feedback on the e-Learning course, including content and useability, and the appropriateness of introducing this training into post-secondary ECE curricula. Your view of the modules is valuable to improve the delivery and content prior to integration into ECE curricula.

There are no right or wrong answers. Everything discussed today will be kept confidential, and your name will be removed from the transcripts and publications. In order to ensure we accurately capture your responses, the interview will be recorded and transcribed. As a reminder, you can withdraw your interview transcript at any time prior to data analysis, and your participation in this interview is separate from your participation in the e-Learning course and surveys. Video will remain off for the interview.

Do you have any questions before we start?

Do you consent to participate?

Do you consent to the publication of direct quotes from this interview transcript?

First, we are going to start with some demographic information. I have created a poll in Zoom that should pop up momentarily. You are welcome to fill out your responses and click submit, but feel free to skip any questions if you are not comfortable sharing this information. We are collecting some of this information again, because your survey responses cannot be linked to your interview data, and we will be describing our interview sample in our publication. The polls function within Zoom only allows for multiple choice questions, so there may be instances where you will be asked if you are comfortable verbally sharing your responses to certain questions.

1. What are your thoughts on using an e-Learning platform to deliver this training?
  - Please expand.
  - Is this method of delivery preferable to in-person education?
  - In what ways did this platform of delivery impact your learning?
2. What characteristics of the e-Learning course did you enjoy the most (this refers to the visual appeal, functioning, navigation, course elements, etc.)?
  - What made those parts/characteristics so enjoyable?
  - What are some examples of these?
  - Tell me more about that.
3. What content in the e-Learning course did you find most useful to you as a (future) early childhood educator?
  - What made it so useful?
  - What are some examples?
4. What content in the e-Learning course did you find least useful to you as a (future) early childhood educator?
  - What about this content did you not find useful?
  - What are some examples?
  - How do you think this content could be delivered differently to make it more useful?
5. What characteristics of the e-Learning course delivery (text, audio, animations, videos, external links, knowledge checks/assessments) do you feel were most beneficial for supporting your learning?
  - What made them so beneficial?
  - What are some examples?
6. What characteristics of the e-Learning course delivery do you feel were least beneficial for supporting your learning?
  - What made them so unbeneficial?
  - What are some examples?
  - How do you think this aspect of the training could be tweaked so that it is more conducive to supporting your learning?
7. Do you think the knowledge assessments at the end of each module were appropriate in complexity?
8. What challenges (if any) did you experience when completing the e-Learning course?
  - Please expand.
  - In what ways did this impact your learning?
9. What solutions did you undertake to deal with these challenges?
  - Please expand.
  - Tell me more about that.
  - How much time and effort did these solutions require?
10. Do you have any suggestions that would improve the e-Learning course?
11. What has been your overall experience with the TEACH intervention?
  - How ‘effective’ would you consider this training in increasing early childhood educators’ knowledge and confidence to promote physical activity in childcare settings?
  - How ‘effective’ would you consider this training in increasing early childhood educators’ knowledge and confidence to minimize prolonged sedentary behaviour in childcare settings?
  - Approximately what percentage of the course content was new to you?
  - How important would you consider this e-Learning training to be for [ECE students/early childhood educators]?
    - How does this e-Learning course align with early childhood educators’ perspectives and beliefs?
    - Did this course change your perspectives regarding the importance of appropriate physical activity experiences in early learning settings?
      - In what ways?
      - Do you think you will use any of the ideas from the e-Learning course in your programming?
  - Was this e-Learning course relevant to your personal experience with childcare programming?
    - How do you think the course could be adapted to better align with your experiences?
  - How well do you see this course integrating into post-secondary ECE programs?
    - Do you think ECE students would be receptive to this type of education in their program?
  - ECE students: How well did this training complement your ECE training?
  - ECEs: How much did this course differ from other professional learning courses you have taken?
    - What elements of this e-Learning course were better than previous e-Learning courses you have taken?
    - What elements of this e-Learning course did not live up to previous e-Learning courses you have taken?
  - How receptive were your classmates/colleagues to this intervention?
  - Do you have anything else to add?

Sample Interview Guide for ECE Instructors

Thank you for volunteering to participate in this interview. Today we will discuss your thoughts and experiences about facilitating the implementation of the TEACH intervention; an e-Learning physical activity training program for [Early Childhood Education (ECE) students in Canadian colleges/universities]. Specifically, we are looking to gather your feedback on the e-Learning course, including content and useability, and the appropriateness and feasibility of introducing this training into post-secondary ECE curricula. Your view of the modules is valuable to improve the delivery and content prior to integration into ECE curricula.

There are no right or wrong answers. Everything discussed today will be kept confidential, and your name will be removed from the transcripts and publications. In order to ensure we accurately capture your responses, the interview will be recorded and transcribed. As a reminder, you can withdraw your interview transcript at any time prior to data analysis, and your participation in this interview is separate from your participation in the survey. Video will remain off for the interview.

Do you have any questions before we start?

Do you consent to participate?

Do you consent to the publication of direct quotes from this interview transcript?

First, we are going to start with some demographic information. I have created a poll in Zoom that should pop up momentarily. You are welcome to fill out your responses and click submit, but feel free to skip any questions if you are not comfortable sharing this information. We are collecting some of this information again, because your survey responses cannot be linked to your interview data, and we will be describing our interview sample in our publication. The polls function within Zoom only allows for multiple choice questions, so there may be instances where you will be asked if you are comfortable verbally sharing your responses to certain questions.

1. What are your thoughts on using an e-Learning platform to deliver this training?
  - Please expand.
  - In what ways did this platform of delivery impact your students’ learning?
2. What were the best parts of the e-Learning course?
  - What made those parts/characteristics so beneficial?
  - What are some examples of these?
  - Tell me more about that.
3. What content in the e-Learning course did you find most interesting?
  - What made it so interesting?
  - What are some examples?
4. What content in the e-Learning course did you find least interesting?
  - What made it so uninteresting?
  - What are some examples?
  - How do you think this content could be delivered differently to make it more interesting?
5. What characteristic(s) of the e-Learning course do you feel was/were most beneficial for supporting your students’ learning?
  - What made it/them so beneficial?
  - What are some examples?
6. What characteristic(s) of the e-Learning course do you feel was/were least beneficial for supporting your students’ learning?
  - What made it/them so unbeneficial?
  - What are some examples?
  - How do you think this aspect of the training could be tweaked so that it is more conducive to supporting your students’ learning?
7. What challenges did your students experience when completing the e-Learning course?
  - Please expand.
  - In what ways did this impact your students’ learning?
8. What solutions did your students undertake to deal with these challenges?
  - Please expand.
  - Tell me more about that.
  - How much time and effort did these solutions require?
9. Overall, what has been your overall experience with the TEACH intervention?
  - How ‘effective’ would you consider this training in increasing ECE students’ knowledge and confidence to promote physical activity in childcare settings?
  - How ‘effective’ would you consider this training in increasing ECE students’ knowledge and confidence to minimize prolonged sedentary behaviour in childcare settings?
  - Do you think this e-Learning course aligns with curriculum objectives of your school’s ECE program?
  - How important would you consider this e-Learning training to be for ECE students?
  - How ‘feasible’ would you consider this e-Learning training to implement in post-secondary ECE programs?
    - *If feasible*: How would you integrate this e-Learning course into the curriculum?
    - *If infeasible*: What would you change about this e-Learning course to make it more feasible to implement in post-secondary ECE programs?
  - How receptive were your students to this intervention?
  - Do you have anything else to add?

## Appendix C

**Table A1:** Consolidated Framework for Implementation Research Construct Checklist for the TEACH Study.

	Consolidated Framework for Implementation Research Construct Checklist			
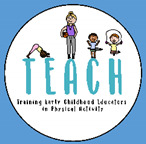
Construct	Short Description	Source Determining Fulfillment	Was the Construct Fulfilled?	How the Construct Was/Will Be Fulfilled
I. INTERVENTION CHARACTERISTICS				
A. Intervention Source	Perception of key stakeholders about whether the intervention is externally or internally developed.	Delphi Study (Bruijns et al., 2020)	Yes	The e-Learning course was developed in collaboration with ECE experts.
B. Evidence Strength & Quality	Stakeholders’ perceptions of the quality and validity of evidence supporting the belief that the intervention will have desired outcomes.	Needs Assessment (Bruijns et al., 2019)	Yes	ECE stakeholders (college students, staff) were involved in this study, which determined that ECE students’ physical activity self-efficacy was higher if they had completed physical activity training.
C. Relative Advantage	Stakeholders’ perception of the advantage of implementing the intervention versus an alternative solution.	Delphi Study (Bruijns et al., 2020)	Yes	ECE experts communicated that this type of training was important for ECE students and supported (and helped with) the creation of content for the e-Learning course.
D. Adaptability	The degree to which an intervention can be adapted, tailored, refined, or reinvented to meet local needs.	e-Learning Course Development (Summer 2020)	--	ECE students will be able to complete the e-Learning course at their own pace within a 4-week timeframe. The course itself will take ~3 h to complete.
E. Trialability	The ability to test the intervention on a small scale in the organization, and to be able to reverse course (undo implementation) if warranted.	Pilot Study (Winter 2021)	--	The intervention will be trialed with one college/university (~50 ECE students) as well as ~20 early childhood educators prior to large-scale implementation.
F. Complexity	Perceived difficulty of implementation, reflected by duration, scope, radicalness, disruptiveness, centrality, and intricacy and number of steps required to implement.	Program Evaluation Survey, Interviews (Winter 2021, 2022)	--	ECE students and instructors will be asked about the perceived ease of completion (students) or implementation (instructors).
G. Design Quality & Packaging	Perceived excellence in how the intervention is bundled, presented, and assembled.	Program Evaluation Survey, Interviews (Winter 2021, 2022)	--	ECE students and instructors will be asked about their perceptions of the e-Learning course’s design quality and presentation.
H. Cost	Costs of the intervention and associated with implementing the intervention including investment, supply, and opportunity costs.	Government Funding (2019–2023)	Yes	Once created, the e-Learning course will only require webhosting (incurred for this project by the research team). For sustainability of the training, students or colleges may be required to pay a small fee to use the service, unless additional funds become available to the research team.
II. OUTER SETTING				
A. Patient Needs & Resources	The extent to which patient needs, as well as barriers and facilitators to meet those needs, are accurately known and prioritized by the organization.	e-Learning Course Development (Summer 2020)	--	ECE students and instructors will be consulted during e-Learning course development to ensure online learning needs are met.
B. Cosmopolitanism	The degree to which an organization is networked with other external organizations.	Ministry of Colleges and Universities	Yes	Each college/university is nested within their province’s Ministry of Colleges and Universities.
C. Peer Pressure	Mimetic or competitive pressure to implement an intervention; typically because most or other key peer or competing organizations have already implemented or are in a bid for a competitive edge.	Recruitment (Summer/Fall 2021)	--	The participation of other colleges and universities is likely to encourage further participation, as this unique training will give programs a competitive edge by being the first to offer the learning opportunity to students.
D. External Policy & Incentives	A broad construct that includes external strategies to spread interventions, including policy and regulations (governmental or other central entity), external mandates, recommendations and guidelines, pay-for-performance, collaboratives, and public or benchmark reporting.	Knowledge Mobilization (2022–2023)	--	Upon completion of the study, knowledge mobilization efforts will be aimed at college and university ECE curriculum experts, childcare organizations, and provincial policymakers to encourage the adoption of this training, as required, for the ECE profession.
III. INNER SETTING				
A. Structural Characteristics	The social architecture, age, maturity, and size of an organization.	Recruitment of College/University ECE Programs	Yes	The relatively new regulation of the early childhood educator profession has prompted the introduction of more college and university ECE programs. As such, ECE curricula are changing, and reviewed regularly to accommodate new research and foci. The nesting of ECE programs in larger, well-established academic institutions ensures resources are available for these changes.
B. Networks & Communications	The nature and quality of webs of social networks and the nature and quality of formal and informal communications within an organization.	ECE Program Faculty and Staff	Yes	ECE program staff and faculty have strong relationships and work collaboratively to provide ECE students with high-quality educational experiences.
C. Culture	Norms, values, and basic assumptions of a given organization.	College and University Reputation	Yes	Colleges and universities are esteemed to provide high-quality educational experiences and are increasingly using online platforms to deliver course content.
D. Implementation Climate	The absorptive capacity for change, shared receptivity of involved individuals to an intervention, and the extent to which use of that intervention will be rewarded, supported, and expected within their organization.			
1. Tension for Change	The degree to which stakeholders perceive the current situation as intolerable or needing change.	Needs Assessment (Bruijns et al., 2019)	Yes	ECE students communicated that they wished to receive more training in physical activity and sedentary behaviour.
2. Compatibility	The degree of tangible fit between meaning and values attached to the intervention by involved individuals, how those align with individuals’ own norms, values, and perceived risks and needs, and how the intervention fits with existing workflows and systems.	Needs Assessment (Bruijns et al., 2019) Delphi Study (Bruijns et al., 2020)	Yes	The e-Learning course we are developing addresses the gaps in content revealed in the needs assessment by ECE students. The Delphi study with ECE experts highlighted that the content developed for the course aligns with ECE curriculum objectives.
3. Relative Priority	Individuals’ shared perception of the importance of the implementation within the organization.	Delphi Study (Bruijns et al., 2020) Program Evaluation Survey (Winter 2021, 2022)	--	ECE experts communicated that this training was important for ECE students to receive in their program. This will also be explored via the program evaluation.
4. Organizational Incentives & Rewards	Extrinsic incentives such as goal-sharing awards, performance reviews, promotions, and raises in salary, and less tangible incentives such as increased stature or respect.	e-Learning Course Certificate	--	ECE students will receive a certificate of completion for the e-Learning course, which they can put on their resume for increased hirability upon graduation.
5. Goals and Feedback	The degree to which goals are clearly communicated, acted upon, and fed back to staff, and alignment of that feedback with goals.	Communication with ECE Instructors	--	ECE instructors implementing the intervention in their classroom will receive regular progress updates from the research team on their students’ e-Learning course completion rates.
6. Learning Climate	A climate in which: a) leaders express their own fallibility and need for team members’ assistance and input; b) team members feel that they are essential, valued, and knowledgeable partners in the change process; c) individuals feel psychologically safe to try new methods; and d) there is sufficient time and space for reflective thinking and evaluation.	Communication with ECE instructors	--	ECE instructors will act as partners in the intervention process. Adequate time will be given for ECE students to complete the e-Learning course, which will allow students to complete it at their own pace and give ECE instructors the ability to attend to student questions and concerns.
E. Readiness for Implementation	Tangible and immediate indicators of organizational commitment to its decision to implement an intervention.			
1. Leadership Engagement	Commitment, involvement, and accountability of leaders and managers with the implementation.	Communication with ECE Program Staff, Website Metrics	--	Researchers will be in constant communication with ECE program staff and instructors regarding their students’ completion rates of the e-Learning modules to hold them accountable.
2. Available Resources	The level of resources dedicated for implementation and on-going operations, including money, training, education, physical space, and time.	e-Learning Course Development (Summer 2020), ECE Instructor Support	--	The e-Learning course will be designed to take ~4 h to complete – a reasonable time requirement to integrate into pre-existing ECE courses. The course will be able to be accessed via mobile phone, tablet, laptop, or desktop, offering flexibility for ECE students. The course will initially be free of cost, and course instructors will be given a brief tutorial on how to use the e-Learning course so they can help their students.
3. Access to Knowledge & Information	Ease of access to digestible information and knowledge about the intervention and how to incorporate it into work tasks.	Communication with ECE Program Staff and Instructors	--	The research team will be readily available to answer any questions from participating ECE programs regarding program implementation.
IV. CHARACTERISTICS OF INDIVIDUALS				
A. Knowledge & Beliefs about the Intervention	Individuals’ attitudes toward and value placed on the intervention as well as familiarity with facts, truths, and principles related to the intervention.	Demographics Survey (Winter 2022)	--	ECE students’ perceived value placed on this type of training will be measured prior to implementation.
B. Self-efficacy	Individual belief in their own capabilities to execute courses of action to achieve implementation goals.	Self-Efficacy Survey (Winter 2022)	--	ECE students’ perceived self-efficacy to successfully complete the e-Learning course will be measured prior to implementation.
C. Individual Stage of Change	Characterization of the phase an individual is in, as he or she progresses toward skilled, enthusiastic, and sustained use of the intervention.	Demographics Survey, Program Evaluation Survey (Winter 2022)	--	ECE students’ motivation to learn about physical activity and sedentary behaviour will be measured pre- and post-intervention, as well as their likelihood of using the knowledge they gained in their future profession.
D. Individual Identification with Organization	How individuals perceive the organization, and their relationship and degree of commitment with that organization.	Demographics Survey (Winter 2022)	--	ECE students’ level of commitment to their studies will be measured as part of participant demographics.
E. Other Personal Attributes	Other personal traits such as tolerance of ambiguity, intellectual ability, motivation, values, competence, capacity, and learning style.	Demographics Survey, Behavioural Intention Survey, Self-Efficacy Survey (Winter 2022)	--	ECE students’ motivation to learn about physical activity and sedentary behaviour, as well as their own physical activity levels and self-efficacy to use e-Learning platforms will be measured prior to implementation.
V. PROCESS				
A. Planning	The degree to which a scheme or method of behaviour and tasks for implementing an intervention are developed in advance, and the quality of those schemes or methods.	Communication with ECE Program Staff and Instructors	--	Early recruitment of colleges and universities will allow plenty of time for the research team to communicate with ECE program staff and instructors regarding timelines, surveys, and logistics of the e-Learning course.
B. Engaging	Attracting and involving appropriate individuals in the implementation and use of the intervention through a combined strategy of social marketing, education, role modeling, training, and other similar activities.			
1. Opinion Leaders	Individuals in an organization who have formal or informal influence on the attitudes and beliefs of their colleagues with respect to implementing the intervention.	Recruitment of ECE Programs (Summer/Fall 2021)	--	ECE programs will be recruited to participate in the intervention study, and program staff will act as opinion leaders who will manage implementation by course instructors.
2. Formally Appointed Internal Implementation Leaders	Individuals from within the organization who have been formally appointed with responsibility for implementing an intervention as coordinator, project manager, team leader, or other similar role.	Recruitment of ECE Instructors (Fall 2021)	--	ECE course instructors will be recruited to implement the intervention with students in their class.
3. Champions	“Individuals who dedicate themselves to supporting, marketing, and ‘driving through’ an [implementation]”, overcoming indifference or resistance that the intervention may provoke in an organization.	Research Team, Recruitment of ECE Student Champions (Fall 2021)	--	Research team members in each province will help champion the intervention, while ECE students in intervention classrooms will be recruited to promote their classmates’ completion of the e-Learning course.
4. External Change Agents	Individuals who are affiliated with an outside entity who formally influence or facilitate intervention decisions in a desirable direction.	Communication with Stakeholder Groups (Ongoing)	--	The research team has been in communication with physical activity and early childhood organizations to include their content and promote our research project nationally.
C. Executing	Carrying out or accomplishing the implementation according to plan.	Website Metrics (Winter 2022)	--	Dose received will be calculated by ECE students’ completion rates of each module within the e-Learning course. Average quiz scores will be calculated for each module to capture the extent of students’ learning.
D. Reflecting & Evaluating	Quantitative and qualitative feedback about the progress and quality of implementation accompanied with regular personal and team debriefing about progress and experience.	Communication with ECE Programs, Program Evaluation Survey, Interviews (Winter 2022)	--	Researchers will be in constant communication with ECE programs about progress. Program Evaluation Surveys and Interviews with ECE students and instructors will capture their experiences with the e-Learning course and its implementation.
